# Unraveling the impact of *Lactobacillus* spp. and other urinary microorganisms on the efficacy of mirabegron in female patients with overactive bladder

**DOI:** 10.3389/fcimb.2022.1030315

**Published:** 2022-11-14

**Authors:** Zhipeng Zhou, Yifeng Qiu, Kun Li, Qi Sun, Ming Xie, Pengcheng Huang, Yao Yu, Benlin Wang, Jingwen Xue, Zhangrui Zhu, Zhengyuan Feng, Jie Zhao, Peng Wu

**Affiliations:** ^1^ Department of Urology, Nanfang Hospital, Southern Medical University, Guangzhou, China; ^2^ Department of Urology, Jinshan Branch of Fujian Provincial Hospital, Fuzhou, China; ^3^ Department of Urology, Fujian Provincial Clinical Medical College, Fujian Medical University, Fuzhou, China; ^4^ School of Pharmaceutical Sciences, Southern Medical University, Guangzhou, China

**Keywords:** female urinary microbiome, overactive bladder, mirabegron, *Lactobacillus*, biomarker

## Abstract

**Objective:**

Overactive bladder (OAB) is a disease that seriously affects patients’ quality of life and mental health. To address this issue, more and more researchers are examining the relationship between OAB treatment and urinary microecology. In this study, we sought to determine whether differences in treatment efficacy were related to microbiome diversity and composition as well as the abundance of specific genera. Machine learning algorithms were used to construct predictive models for urine microbiota-based treatment of OAB.

**Methods:**

Urine samples were obtained from 64 adult female OAB patients for 16S rRNA gene sequencing. Patients’ overactive bladder symptom scores (OABSS) were collected before and after mirabegron treatment and patients were divided into effective and ineffective groups. The relationship between the relative abundance of certain genera and OABSS were analyzed. Three machine learning algorithms, including random forest (RF), supporting vector machine (SVM) and eXtreme gradient boosting (XGBoost) were utilized to predict the therapeutic effect of mirabegron based on the relative abundance of certain genera in OAB patients’ urine microbiome.

**Results:**

The species composition of the two groups differed. For one, the relative abundance of *Lactobacillus* was significantly higher in the effective group than in the ineffective group. In addition, the relative abundance of *Gardnerella* and *Prevotella* in the effective group was significantly lower than in the ineffective group. Alpha-diversity and beta-diversity differed significantly between the two groups. LEfSe analysis revealed that *Lactobacillus* abundance increased while *Prevotella* and *Gardnerella* abundance decreased in the effective group. The *Lactobacillus* abundance ROC curve had high predictive accuracy. The OABSS after treatment was negatively correlated with the abundance of *Lactobacillus*, whereas the relationship between OABSS and *Prevotella* and *Gardnerella* showed the opposite trend. In addition, RF, SVM and XGBoost models demonstrated high predictive ability to assess the effect of mirabegron in OAB patients in the test cohort.

**Conclusions:**

The results of this study indicate that urinary microbiota might influence the efficacy of mirabegron, and that *Lactobacillus* might be a potential marker for evaluating the therapeutic efficacy of mirabegron in OAB patients.

## Introduction

It is well known that homeostasis in the human body is inextricably linked to the microbial community, and the urinary system is no exception. Expanded quantitative urine culture and molecular biology techniques have been used to disprove the “sterile urine” paradigm in healthy people (Wolfe et al., 2012; Aragón et al., 2018). Recent studies have shown that the urinary system has a unique urinary microbiome that influences the development and progression of urinary system disorders such as urinary tract infection, bladder cancer, prostate cancer, interstitial cystitis, chronic pelvic pain syndrome and overactive bladder (OAB) (Magistro and Stief, 2019; Markowski et al., 2019; Neugent et al., 2020; Huang et al., 2021).

The International Continence Society (ICS) defines OAB as “urgent urination with or without urinary incontinence (UI), usually increased daytime urination and nocturnal urination” (Abrams et al., 2002). The prevalence of OAB in the Asia–Pacific region is estimated at 20.8% and is known to increase with age (Chow et al., 2018). Known as a “social cancer,” OAB seriously impacts not only patients’ quality of life, but also their mental health. Existing OAB treatment options include behavioral training, drug intervention, detrusor injection and electrical stimulation of sacral or tibial nerves, but the curative effect of these options is limited. In recent years, with the growing advances and popularity of microbial research, the microbial community has received extensive attention. Available literature has reported on the presence of diverse types of bacteria in the female urinary system in OAB patients and controls (Curtiss et al., 2017). Other studies also have found a decrease in the prevalence of *Lactobacillus* in patients with bladder symptoms (Fouts et al., 2012). Meanwhile, certain characteristics of the urinary microbiome have been strongly associated with the severity of OAB symptoms (Li et al., 2022). Our previous studies suggested that the presence of OAB may be associated with a decrease in the diversity and abundance of the urine microbiome and offered important implications for guiding the treatment of OAB (Wu et al., 2017).

Mirabegron is currently widely used as a first-line drug in OAB treatment. A cohort study of 568 patients on the efficacy of mirabegron showed that the use of mirabegron and tamsulosin was more effective than the placebo in patients with lower urinary tract symptoms (LUTS) and OAB symptoms (Kakizaki et al., 2020). Interestingly, another study reported that treatment with mirabegron alone was less effective than treatment with solifenacin plus mirabegron (Kelleher et al., 2018). Across clinical observations and literature reports, the efficacy of mirabegron varies widely. To date, it is unknown whether differences in efficacy are related to urinary system microbiota.

While many studies have reported on the relationship between urinary microbiota and OAB, the roles of certain bacteria and the ability of machine learning algorithms to predict the efficacy of mirabegron-based OAB therapy have not yet been explored. In this study, we sought to identify differences in urine microbiota between two groups based on mirabegron’s efficacy and elucidate the potential role of urine microbiota composition as a biomarker for OAB treatment. In addition, we sought to develop a predictive model for OAB based on machine learning algorithms.

## Material and methods

### Subject recruitment and research design

This research was conducted in Nanfang Hospital and approved by the Institutional Review Board of Nanfang Hospital (reference number: NFEC-2020-123). Oral and written consent to the study was given to participants. A total of 82 adult female participants from ages 18 to 63 years old with OAB were recruited from an outpatient clinic in Nanfang Hospital from June 2020 to December 2020. Patients with any one of the following conditions were ineligible: urinary tract infection, antibiotic use within the past 30 days, long-term indwelling catheterization or intubation for up to 2 weeks, various cystitis, urinary urogenital cancer, urinary tract stones, neurogenic bladder, pelvic organ prolapse, pelvic radiotherapy, pregnancy and reluctance to participate in the study.

Of the 82 adult (≥ 18 years of age) female patients diagnosed with OAB, 12 were ineligible due to low levels of extracted bacterial DNA and six failed to follow up after 3 months of drug treatment. Ultimately, a total of 64 patients from ages 18 to 63 years old were included, and 64 urine samples were collected by catheterization.

**Figure 1:**
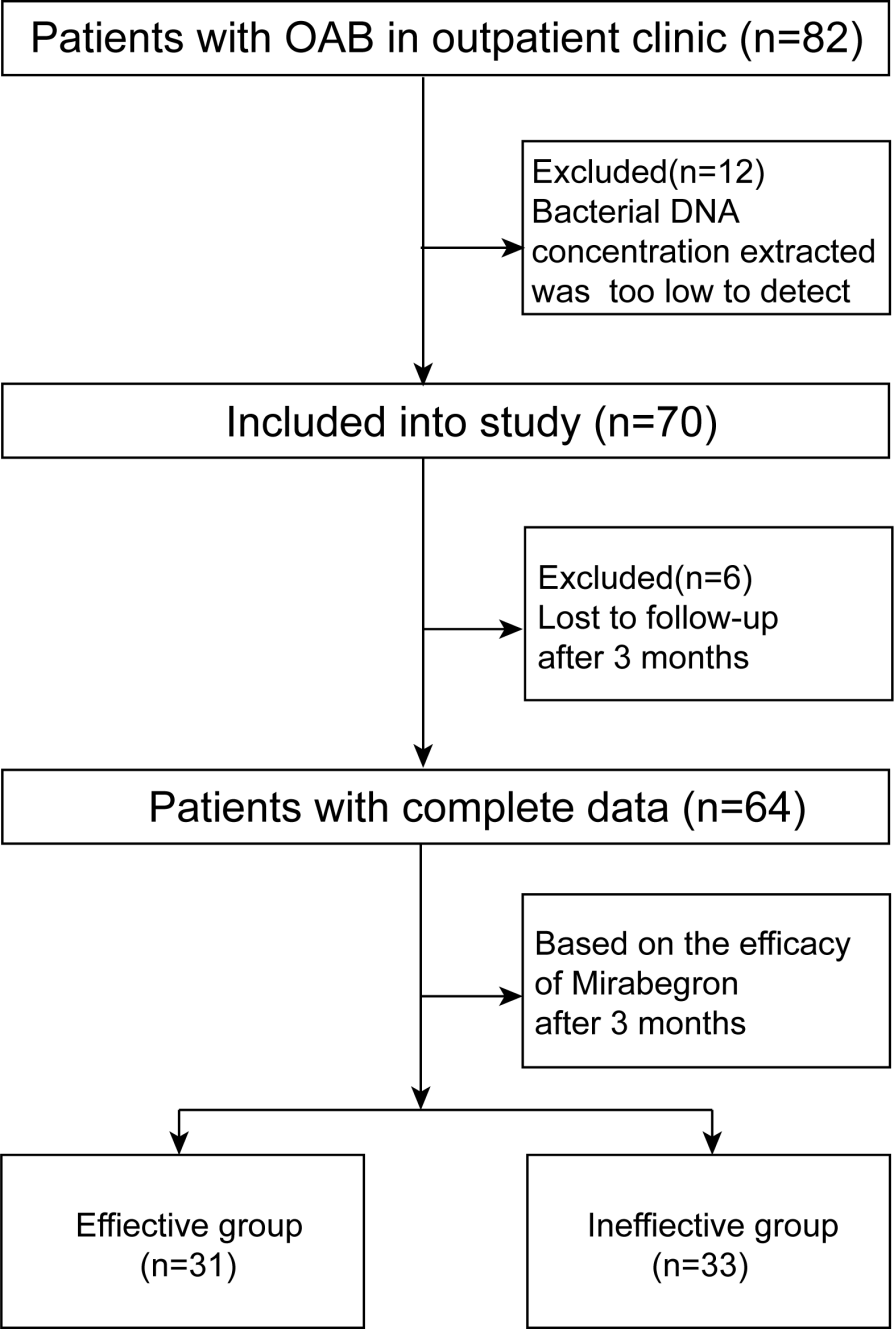
Flowchart of this study.

Overactive bladder symptom scores (OABSS) were completed by every participant at the beginning of the clinic (Homma et al., 2006). After 3 months, according to the efficacy of mirabegron, participants were divided into an effective group and an ineffective group. At the end of the treatment, it was required that OABSS be completed again. The patient recruitment process is displayed in **Figure 1**.

### Urine collection

Due to differences in microbiota between the bladder and urethra and a desire to avoid distal urethral, vulval or vaginal contamination, 50 ml of urine samples were collected by catheterization (Hiergeist and Gessner, 2017; Pohl et al., 2020). Half of the sample (25 ml) was used for culturing to rule out UTI. The remaining samples (25 ml) were kept at 4 °C and transferred to the laboratory within an hour for immediate centrifugation for 10 min at 16,000 g. The supernatants were stored at −80°C until further processing.

### DNA isolation and 16S rRNA gene sequencing

In order to maintain high purity, microbial genomic DNA was extracted from urine samples using a DNeasy Blood and Tissue Kit (Qiagen, Germany), strictly following the manufacturer’s protocol. A Nanodrop ND-1000 spectrophotometer (Thermo Electron Corporation, USA) was used to measure the concentration of extracted DNA. Taq PCR Core Kit (Qiagen, Germany) were used to amplify 16S rDNA isolated from the urine specimens with primers targeting V3-V4 regions (314F: 5′-NCCT ACGGGNGGCWGCAG-3′; 805R: 5′NGACTACHVGGGTATCTAATCC-3′) (Chen et al., 2022). PCR cycling conditions were set based on the manufacturer’s instructions (initial denaturation: 3 min at 94°C; 3-step cycling [cycling number: 30]: Denaturation [0.5min at 94°C], annealing [0.5min at 55°C], Extension [1 min at 72°C]; Final extension: 10 min at 72°C). PCR products were purified by using the Qiaquick PCR purification kit (Qiagen, Germany). 16S rRNA sequencing was performed on the Illumina MiSeq platform (Illumina, Inc, USA) and 250-bp paired-end reads were obtained. The primers used for PCR amplification contained adapters for MiSeq sequencing and dual-indexed barcodes to ensure that the PCR products were able to be sequenced directly. QIIME quality trimming was performed as previously described (Fadrosh et al., 2014). Data from this study are stored in the NCBI SRA database (PRJNA661243).

## Bioinformatics

In order to maintain high quality sequences and remove adapters pollution, the original gene sequences readings were filtered using the wrapper package Quantitative Insights Into Microbial Ecology (QIIME, version 1.8) (Caporaso et al., 2010). With the assistance of UPARSE, the filtered sequences were clustered into operational taxonomic units (OTUs) at 97% similarity levels. (Edgar, 2013). The Ribosomal Database Project Classifier was then used for OTU taxonomic assignment using the SILVA database.

Alpha-diversity was obtained using Chao-1, Shannon and Simpson indices. Chao 1 represented the species richness of the urine microbiome, while Shannon and Simpson diversity both captured species richness and evenness. The statistical significance of differences in alpha diversity between the two groups was evaluated using the Wilcoxon rank-sum test. Changes in urine microbiota community structure were expressed as beta diversity and calculated by principal coordinate analysis (PCoA) using Bray–Curtis, weighted UniFrac and unweighted UniFrac distance. The statistical significance of differences in beta diversity between effective and ineffective groups was evaluated using PERMANOVA and PERMDISP tests. Linear discriminant analysis effect size (LEfSe) was employed to evaluate differentially abundant bacterial genera between the two groups on the MicrobiomeAnalyst platform (Chong et al., 2020). The logarithmic LDA score value for discriminative features was 2.0. Finally, the area under the receiver operating characteristic (ROC) curve (AUC) was used to evaluate predictive accuracy.

### Machine learning algorithms

First, all samples were divided into training (70%, *n* = 46) and test sets (30%, *n* = 18) with the “caret” software package. The three machine learning algorithms random forest (RF), supporting vector machine (SVM) and eXtreme gradient boosting (XGBoost) were utilized to predict the therapeutic effect of mirabegron based on the relative abundance of certain genera in the OAB patient urinary microbiome. Ten-fold cross-validation was performed to obtain the optimal parameters and improve the performance of the three models. The parameters for the XGBoost model were as follows: nrounds = 75, max_depth = 2, eta = 0.01, gamma = 0.5, colsample_bytree = 1, min_child_weight = 1 and subsample = 0.5. For the RF model, the following parameters were applied: entree = 500 and try = 28. For the SVM model, the following parameters were utilized: kernel = “radial,” gamma = 0.01 and cost = 1. All machine learning analyses were conducted using “Random Forest,” “e1071,” and “XGBoost” packages.

## Statistical analysis

The normally distributed continuous variables were described as means and standard deviations (SD), while the non-normally distributed continuous variables were reported as medians with interquartile ranges (Q1–Q3). Classified variables were expressed in frequencies and percentages. Continuous variables were compared with a *t*-test or Mann-Whitney *U* test and classified variables were compared with a Chi-square or Fisher’s exact test. Statistical analyses were performed using SPSS software (version 23, IBM Corp., USA). Differences were considered significant when the *p*-value was less than 0.05.

## Results

### Demographic characteristics of subjects

It is known that many factors, such as age, body mass index (BMI) and history of pelvic surgery, influence the occurrence of OAB and alter urinary microbiota. To minimize the influence of these factors on this study’s results, the demographic characteristics of the two cohorts were matched. Demographic characteristics, including age, BMI, past pregnancy, prior estrogen use, history of pelvic surgery, hypertension, diabetes, menopausal status and OABSS, are presented in **Table 1**. As is shown in the table, there were no statistically significant differences in age (*p* = 0.867), BMI (*p* = 0.936), past pregnancy (*p* = 0.885), menopause (*p* = 0.536), prior estrogen use (*p* = 0.073), diabetes (*p* = 1.000), hypertension (*p* = 0.493), and history of pelvic surgery (*p* = 0.485). Notably, OABSS means in the effective and ineffective groups were 7.16 and 7.94, respectively, and there was no significant difference between the two groups (*p* = 0.278).

**Table 1:** Comparisons of characteristics between effective group and ineffective group.

Characteristic	Effective group	Ineffective group	p-Value
Age	37.90 (1.82)	37.42 (2.17)	0.867
BMI	21.36 (0.51)	21.31 (0.53)	0.936
Ever pregnant	23 (74.2)	25 (75.8)	0.885
Menopause	28 (90.3)	27 (81.8)	0.536
Previous estrogen use	0 (0)	5 (15.1)	0.073
Diabetes	1 (3.2)	2 (6.0)	1.000
Hypertension	0 (0)	2 (6.0)	0.493
Pelvic surgery	7 (41.2)	10 (58.8)	0.485
OABSS	7.16 (0.48)	7.94 (0.52)	0.278

BMI, Body mass index; OABSS, Overactive Bladder symptom Scale.

Data are showed as mean (SD) or median (interquartile ranges) for or n (%) for counts. continuous variables.

### Sequencing data and microbial diversity

**Figure 2:**
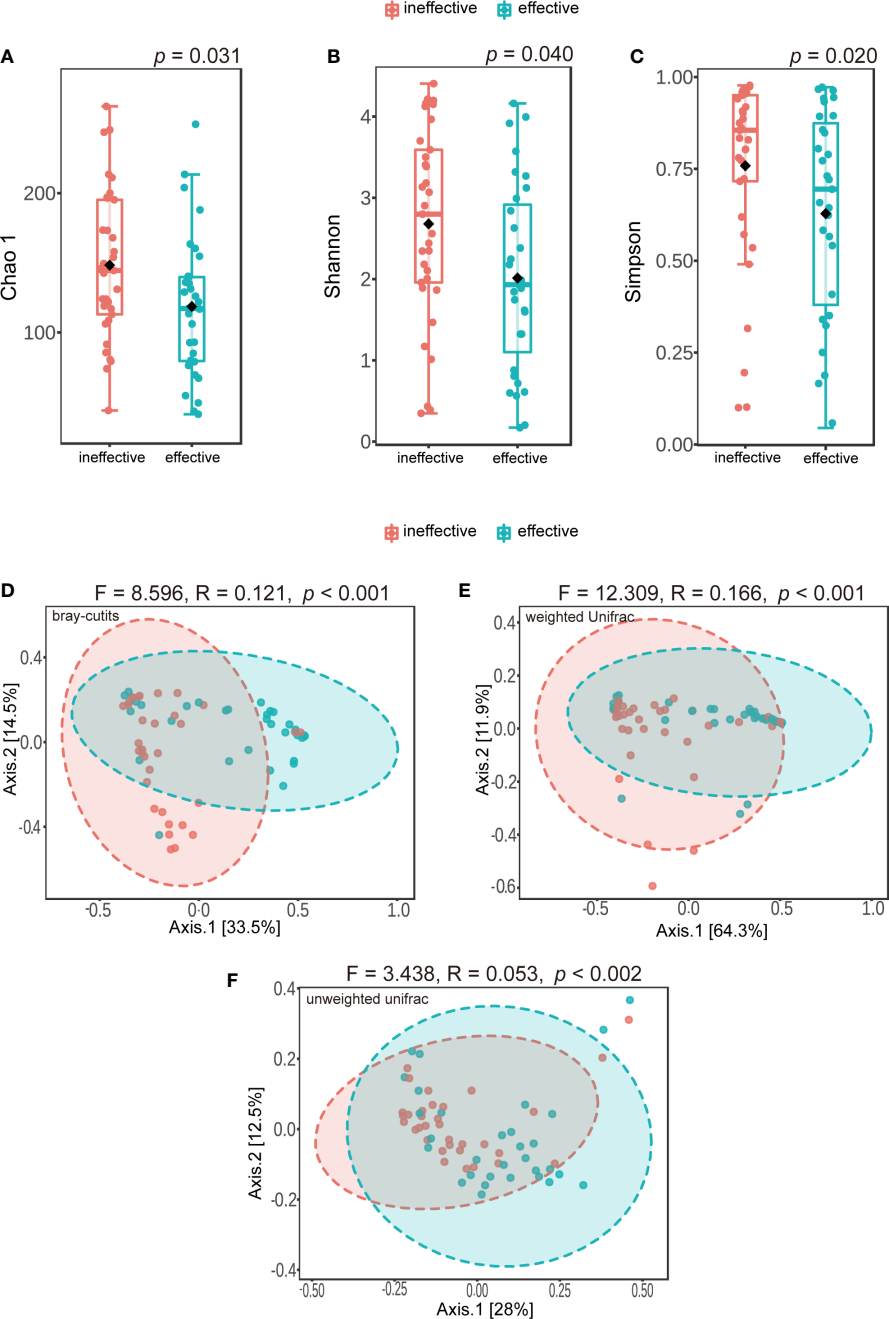
Alpha-diversity and principal coordinate analysis for urinary microbiomes between ineffective group and effective group. **(A)** Chao 1 **(B)**; Shannon index **(C)**; Simpson index **(D)**; Principal coordinate analysis plot of the urinary microbiome based on bray-curits **(E)**; or weighted Unifrac **(F)**; or unweighted Unifrac.

A total of 4,305,495 reads and 4,977 OTUs were obtained from 64 urine samples. All of these data were sequenced using an Illumina MiSeq sequencer. We found some meaningful differences in alpha diversity between the two groups, including differences in Chao 1 (*p* = 0.031) (**Figure 2A**), the Shannon index (*p* = 0.040) (**Figure 2B**), and the Simpson index (*p* = 0.020) (**Figure 2C**). Differences in microbiota composition between groups were evaluated with beta diversity, which was calculated using weighted and unweighted UniFrac based on Bray–Curtis distance metrics, and visualized using PCoA. We found significant differences in Bray–Curtis (F = 8.596, R-squared = 0.121, *p* < 0.001) (**Figure 2D**), weighted UniFrac (F = 12.309, R-squared = 0.166, *p* < 0.001) (**Figure 2E**) and unweighted UniFrac distances (F = 3.438, R-squared = 0.053, *p* < 0.002) (**Figure 2F**). However, when using PERMDISP, we did not find significant differences in Bray–Curtis (F = 3.392, *p* = 0.070), weighted UniFrac (F = 0.095, *p* = 0.759) and unweighted UniFrac distances (F = 2.75, *p* = 0.102) (**Supplementary Table 1**).

### Comparison of urine microbiota distribution between groups

**Figure 3:**
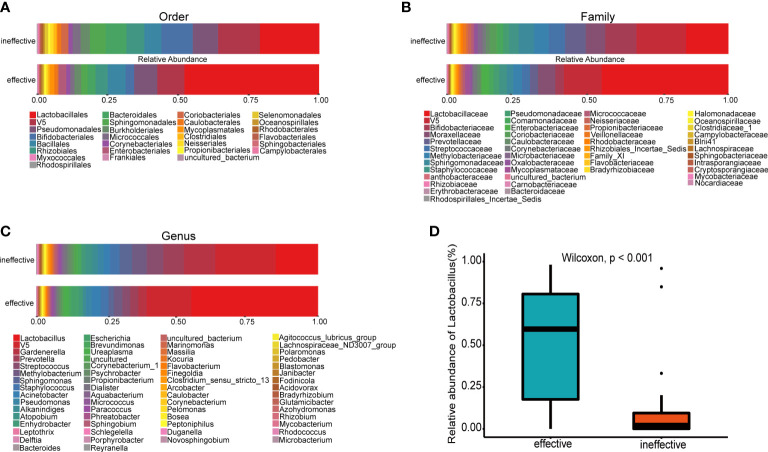
Histogram of species distribution at different levels and comparison of relative abundance of Lactobacillus between the effective and ineffective groups. **(A)** Order **(B)**; Family **(C)**; Genus **(D)**; Comparison of relative abundance of *Lactobacillus* at genus level.

The urine microbial community from all samples displayed apparent differences at several taxonomic levels. Urine samples from the effective group were dominated by the order Lactobacillales (**Figure 3A**), family Lactobacillaceae (**Figure 3B**) and genus *Lactobacillus* (**Figure 3C**). At the genus level, the relative abundance of *Lactobacillus* in the effective group was significantly higher than that in the ineffective group (**Figure 3D**). In a genus-level comparison of the relative abundance of bacterial genera, including *Lactobacillus*, *Gardnerella* and *Prevotella* (**Table 2**), the relative abundance of *Lactobacillus* was significantly higher in the effective group than in the ineffective group (p < 0.001). Furthermore, the relative abundance of *Gardnerella* and *Prevotella* in the effective group was significantly lower than that in the ineffective group (*Gardnerella*: *p* = 0.010, *Prevotella: p* = 0.041).

**Table 2:** Comparisons of median relative abundances of bacteria (genus) in effective group and ineffective group.

Relative abundance (%)	Effective group (n = 31)	Ineffective group (n = 33)	*p*-Value
Lactobacillus	59.643 (15.759, 81.173)	2.199 (0.023, 11.269 )	<0.001
Gardnerella	0.000 (0.000, 0.412)	0.596 (0.000, 14.292)	0.010
Prevotella	0.092 (0.000, 0.825)	0.733 (0.000, 2.749)	0.041

Data presented as median [IQR].

IQR, interquartile range.

### Bacterial genera associated with mirabegron therapeutic efficacy

**Figure 4:**
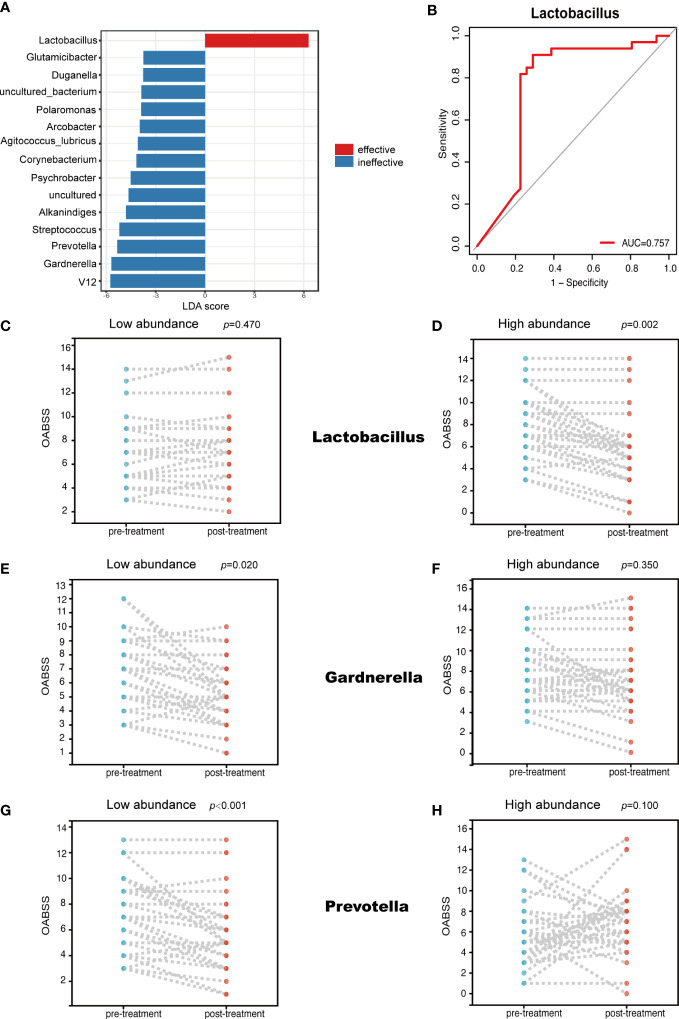
LEfSe analysis and the relationship between the abundance of different genera and OABSS. **(A)** Genera enriched for effective group in red; ineffective group enriched genera in blue. Only genera meeting a linear discriminant analysis score threshold >2.0 are displayed **(B)**; ROC curve showing the ability of *Lactobacillus* to predict efficacy **(C)**; Comparison of OABSS before and after treatment in patients with high abundance of *Lactobacillus* **(D)**; Comparison of OABSS before and after treatment in patients with low abundance of *Lactobacillus* **(E)**; Comparison of OABSS before and after treatment in patients with high abundance of *Gradnerella* **(F)**; Comparison of OABSS before and after treatment in patients with low abundance of *Gardnerella* **(G)**; Comparison of OABSS before and after treatment in patients with high abundance of *Prevotella* **(H)**; Comparison of OABSS before and after treatment in patients with low abundance of *Prevotella*. OABSS, Overactive Bladder Symptom Score (OABSS).

Several genera uniquely enriched in one of the two groups were identified using the LEfSe algorithm (**Figure 4A**). Specifically, the LEfSe analysis revealed that *Lactobacillus* was markedly enriched in the effective group, but on the other hand, *Gardnerella* and *Prevotella* dominated in the ineffective group. Through this analysis, we also found a strong correlation between *Lactobacillus* abundance and mirabegron’s efficacy. When the ROC curve was calculated to evaluate whether *Lactobacillus* could be used to predict mirabegron’s efficacy, *Lactobacillus* abundance (AUC = 0.757) (**Figure 4B**) presented significantly high predictive accuracy. Meanwhile, we found that the relative abundance of *Lactobacillus*, *Gardnerella* and *Prevotella* were associated with mirabegron’s efficacy, which was evaluated based on OABSS. According to the median of genera relative abundances, patients were divided into two clusters based on whether the abundance of a given genus was low or high. In the low-abundance *Lactobacillus* cluster, there was no statistical difference in the extent of OABSS decline (*p* = 0.470) (**Figure 4C**). However, the reverse was true for the high-abundance *Lactobacillus* cluster, with statistically significant differences (*p* = 0.002) (**Figure 4D**), meaning that the high-abundance *Lactobacillus* subset performed better in treatment of OAB with mirabegron. Furthermore, in the low-abundance *Gardnerella* and *Prevotella* clusters, there were statistically significant differences in the degree of OABSS decrease (*p* = 0.020, *p* < 0.001) (**Figures 4E, G**), while in the high-abundance *Gardnerella* and *Prevotella* clusters, the reverse was true (*p* = 0.350, *p* = 0.100) (**Figures 4F, H**). This result suggests that the lower-abundance *Gardnerella* and *Prevotella* subsets were more effective in treatment of OAB with mirabegron.

### Predictive values of machine learning models based on the efficacy of mirabegron

**Figure 5:**
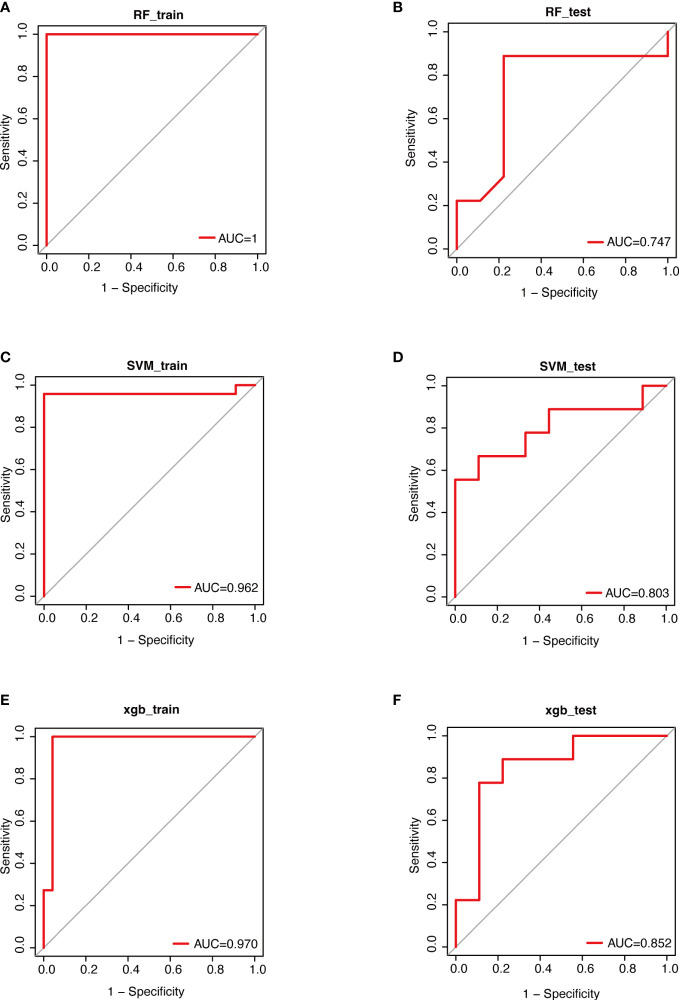
Performance evaluations of therapeutic effects based on 3 algorithms. Data were randomly divided into a training set and a testing set according to 7:3. ROC curves for evaluating the predictive performance of the therapeutic effect. **(A)**; the training set of RF **(B)**; the test set of RF **(C)**; the training set of SVM **(D)**; the testing set of SVM **(E)**; the training set of XGBoost **(F)**; the testing set of XGBoost. RF, Random Forest; SVM, Support Vector Machine; XGBoost, eXtreme Gradient Boosting.

Through aforementioned analyses, we observed that urinary microbiota composition and some specific genera were closely associated with mirabegron’s therapeutic efficacy. Accordingly, we constructed three machine learning models (RF, SVM and XGBoost) based on the relative abundance of all genera in the urinary microbiome. We found that the AUC values of RF, SVM and XGBoost models in the training sets were 1, 0.962 and 0.970, respectively (**Figures 5A, C, E**). Further, the ROC curves revealed relatively good predictive accuracy of the RF, SVM and XGBoost models in the test sets (RF: AUC =0.747, SVM: AUC=0.803, XGBoost: AUC = 0.852; **Figures 5B, D, F**). These results suggest that it may be possible to predict the effectiveness of mirabegron treatment by examining the urinary microbiota of OAB patients. Furthermore, we may be able to enhance the treatment efficacy of mirabegron by modulating urinary microbiota.

## Discussion

The primary goal of treating OAB is to alleviate symptoms and improve quality of life, and medication remains the preferred treatment for the vast majority of patients (Deeks, 2018). Mirabegron has proven effective as a first-line drug in treating OAB in several clinical trials (Herschorn et al., 2013; Kuo et al., 2015; Singh et al., 2021). However, the efficacy of mirabegron varies widely, and the reasons for this variation remain unclear. To our knowledge, while a growing number of studies have reported differences in urine microbiota between OAB and healthy individuals, few studies have considered whether the urine microbiome is associated with mirabegron’s efficacy. In the present study, we described the composition of the urine microbiome of adult female OAB patients, divided into an effective group and ineffective group based on mirabegron’s efficacy, and conducted a series of comparisons. We detected significant differences in both alpha diversity, representing intragroup variation, and beta diversity, representing intergroup variation. Meanwhile, using LEfSe analyses, we found that one genus was enriched and 14 genera were depleted compared to the null group. Furthermore, specific genera associated with mirabegron’s efficacy, such as *Lactobacillus*, *Gardnerella* and *Prevotella*, were identified using the extent to which OABSS declined. In addition, machine learning algorithms, including RF, SVM and XGBoost prediction models, predicted mirabegron’s efficacy against OAB.

Our previous study found that urine samples from patients with severe OAB had higher bacterial diversity (Simpson index) and richness (Chao 1) than those from patients with mild OAB. In this study, the ineffective group had higher Simpson index, Chao 1 and evenness (Shannon index) than the effective group, which is consistent with our earlier study. Meanwhile, a previous study of how bladder bacterial diversity differs in continent and incontinent women revealed that Chao1, Simpson index and Shannon index were all significantly higher in the urinary incontinence cohort compared to the control cohort and positively correlated with the urinary distress inventory (UDI) subscale score (Price et al., 2020). With these reports from the literature, we had reason to speculate that Chao 1, Simpson index and Shannon index reflected OAB severity. In the current study, these indies were higher in the ineffective group than in the effective group, which might imply that non-responders experienced more severe OAB, leading to a poorer outcome for mirabegron treatment. Similarly, the difference in beta diversity, which represented diversity among groups, was statistically significant. These results indicate that the urine microbiome structure of the non-responders is different from that of the responders. In contrast, when Halverson et al. (2022) collected urine samples from 47 participants *via* transurethral catheters and processed samples using expanded quantitative urine culture (EQUC), they found that the differences in alpha diversity were insignificant between responders and non-responders at baseline. However, after 12 weeks of mirabegron treatment, responder urine microbiomes were more diverse than those of non-responders, and the relative abundance of Lactobacillus gasseri was elevated in responders (Halverson et al., 2022). These results are inconsistent with those gathered in our study. We speculate that this discrepancy may come from a number of reasons, including different methods of detecting microbiota, namely, culturing and sequencing; the different times at which specimens were collected; and the possibility that an elevated abundance of *Lactobacillus* was a major factor in responders.

At the order, family and genus levels, the distribution between species was distinct, and the effective group was dominated by *Lactobacillus*. However, in the null group, the abundance of *Gardnerella* and *Prevotella* was higher than that in the effective group, as shown in **Table 2**. Previous studies have found that *Gardnerella* dominates in patients with incontinence, but the abundance of *Lactobacillus* decreases in UUI patients compared to those without UUI (Pearce et al., 2014). Furthermore, *Prevotella*, a Gram-negative anaerobic bacterium that has been identified and isolated from multiple tissues and organs (Tett et al., 2021), has been positively correlated with the severity of OAB symptoms (Li et al., 2022). This research and the present study strongly suggest that abnormalities in species distribution are associated with the extent of OAB and are likely associated with drug efficacy.

To investigate which bacteria might have affected OAB efficacy, we used the LEfSe algorithm to evaluate differences between effective and ineffective groups. We found that, in the effective group, the abundance of one genus increased while that of 14 was reduced. Simultaneously, the ROC curve was calculated to evaluate whether *Lactobacillus* could be used to predict the efficacy of mirabegron, and the abundance of *Lactobacillus* was found to be highly predictive. Changes in the abundance of *Lactobacillus*, *Prevotella* and *Gardnerella* with OABSS were evaluated to assess each genus’ impact on treatment efficacy. The results showed that the decrease in OABSS was more statistically significant in patients with a high abundance of *Lactobacillus* and a low abundance of *Prevotella* and *Gardnerella*. We also found no significant decrease in OABSS in patients with low levels of *Lactobacillus*, and high level of *Prevotella* and *Gardnerella*, suggesting that mirabegron was not effective for these patients. *Lactobacillus* is an anaerobic bacterium that acts as a protective agent by lowering pH, producing antibacterial mixtures and acting as a delivery agent for vaccines or therapeutics (O’Callaghan and O’Toole, 2013). We hypothesize that the acidic environment created by *Lactobacillus* and its antibacterial products increased the affinity of mirabegron, a β3-adrenergic receptor, to enhance mirabegron efficacy. *Gardnerella* is also a facultative anaerobic bacterium linked to bacterial vaginosis. The female urethra’s position adjacent to the vagina may be one reason for the high incidence of OAB in women and the poor efficacy of mirabegron. Finally, *Prevotella* spp. are involved in regulating health homeostasis (Tett et al., 2021). Recent studies have suggested that high levels of *Prevotella* in the mucosa are associated with systemic disease and low levels of systemic inflammation (Larsen, 2017), which may represent another factor affecting the efficacy of mirabegron.

Finally, we employed three machine learning algorithms to predict the efficacy of mirabegron in OAB patients. The results revealed that XGBoost had a higher predictive capability than RF and SVM models. The most important factor in XGBoost’s success is its scalability across all scenarios due to a number of significant system and algorithm enhancements (Hamidi et al., 2021).

Machine learning algorithms have been widely used in oncology research, and with advances in microbiology research, we are the first to use machine learning algorithms to predict OAB efficacy. However, several limitations to this study should be considered. First, the small sample size may warrant caution when interpreting the results. Second, because urine microbiota were only evaluated prior to treatment, and not after treatment, it is impossible to determine how the microbiota changed following mirabegron treatment. The specific mechanisms by which urine microbiota affect the efficacy of mirabegron deserve further study.

## Conclusions

To the best of our knowledge, this is the first study to examine the relationship between urine microbiome characteristics and the efficacy of mirabegron for treating OAB. Our results demonstrated that differences in urine microbiome diversity and composition existed between the effective group and ineffective group. We also found that increasing the abundance of Lactobacillus or decreasing the abundance of Prevotella and Gardnerella may improve the efficacy of mirabegron. Notably, we found that RF, SVM and XGBoost machine learning models performed well in predicting the efficacy of mirabegron based on a relatively abundant genus in OAB patients’ urine microbiome.

## Data availability statement

The datasets presented in this study can be found in online repositories. The names of the repository/repositories and accession number(s) can be found in the article/**Supplementary Material**.

## Ethics statement

This study was approved by the Institutional Research Ethics Committee of the Nangfang Hospital prior to the start of the study (No.NFEC-2020-123) and all participants signed an written informed consent form.

## Author contributions

PW, JZ: conception and design. ZPZ, YQ: data analysis and interpretation, manuscript writing. YY, BW, JX: data management and data analysis. PH, KL, QS, MX: material preparation. ZRZ, ZF: data collection. PW, ZPZ: funding acquisition. All authors contributed and approved the submitted version.

## Funding

This current study was supported by funding from the National Natural Science Foundation of China (grant No. 81870522 and No.82173304); the Natural Science Foundation of Guangdong Province (grant No. 2018A030313148 and No. 2021A1515012262); Startup Fund for Scientific Research, Fujian Medical university (grant No. 2022QH1057). This study also received funding from Astellas Pharma Inc which was not involved in the study design, collection, analysis, interpretation of data and the writing of this article or the decision to submit it for publication.

## Acknowledgments

We acknowledge the hard and dedicated work of all the staff that implemented the intervention and evaluation components of the study.

## Conflict of interest

The authors declare that the research was conducted in the absence of any commercial or financial relationships that could be construed as a potential conflict of interest.

## Publisher’s note

All claims expressed in this article are solely those of the authors and do not necessarily represent those of their affiliated organizations, or those of the publisher, the editors and the reviewers. Any product that may be evaluated in this article, or claim that may be made by its manufacturer, is not guaranteed or endorsed by the publisher.
