# Supplementary material for: Unraveling the impact of Lactobacillus spp. and other urinary microorganisms on the efficacy of mirabegron in female patients with overactive bladder
Source: Front Cell Infect Microbiol. 2022 Nov 14;12:1030315. doi: 10.3389/fcimb.2022.1030315 (PMC9703976; doi:10.3389/fcimb.2022.1030315)
Supplement: Supplementary file 1 [file Table_1.docx]

Supplymentary Table 1 Principal coordinate analysis for urinary microbiomes between ineffective group and effective group by using PERMIDSP method.

| PERMIDSP | F-vaule | *p*-vaule |
| --- | --- | --- |
| Bray-Curtis | 3.392 | 0.070 |
| weighted UniFrac | 0.095 | 0.759 |
| unweighted UniFrac | 2.750 | 0.102 |

*Principal coordinate analysis of the urinary microbiome based on the Bray-Curtis distance metrics, weighted and unweighted UniFrac.*
